# Ilustrations of the Elementary Forms of Disease

**Published:** 1835-01-01

**Authors:** 


*Illustrations of the Elementary Forms of Disease.* By ROBERT CARSWELL, M.D., Professor of Pathological Anatomy in the University of London. Fasciculus VI. *Hemorrhage.*—London, 1834. Folio.

OUR author divides hemorrhage into two great classes, namely, hemorrhage from physical lesions, and hemorrhage from vital lesions. The former again is divided into two genera,—as it may arise from solutions of continuity, or from a mechanical obstacle to the circulation; the latter into three, —as it may arise from a modification of function of the capillaries, from a diseased state of the blood, or from debility. Each of these genera, except the last, is again subdivided.

Dr. Carswell does not agree with those writers who suppose that "paralysis of the *superior* extremities depends on the effusion taking place in the *thalami,* or in the cerebral substance situated on a *level* with and *posterior* to them; and that paralysis of the *inferior* extremities depends on the effusion taking place in the *corpora striata,* or in the cerebral substance situated on a *level* with or *anterior* to them." Nor does he side with those who say "that the *loss of speech,* which not unfrequently accompanies cerebral hemorrhage, depends on the effusion occupying the *anterior lobes* of the brain, a statement which derives still less support from actual observation than the former; for *blood may be effused* in the *anterior lobes* of the brain, *without giving rise to any modification of speech whatever.*"

We must be permitted to observe, that Dr. Carswell's statement, and the one which he oppugns, are by no means irreconcileable; for it is easy to suppose that apoplectic aphonia always depends on effusion in the anterior lobes, and also that this effusion may exist without producing the aphonia. Our author thinks that

"the best established facts regarding the seat of cerebral hemorrhage, and the relation which exists between it and paralysis, are the following:

"1. That the paralysis occupies the side of the body opposite to that of the brain or cerebellum in which the effused blood is situated.

"2. That the paralysis affects only one side of the body when the effused blood is confined to one hemisphere of the brain, or one of the lateral lobes of the cerebellum.

"3. That the paralysis exists on both sides of the body when the hemorrhage has taken place in both hemispheres of the brain, or both lateral lobes of the cerebellum; into the ventricles, the pons Varolii, the medulla oblongata, and on the surface of the brain.

"4. That paralysis of both sides of the body may also take place when the hemorrhage is confined to one hemisphere of the brain, or lateral lobe of the cerebellum, but is so extensive as to produce compression of the opposite hemisphere or lobe."

Dr. Carswell observes, that "as hemorrhage of one of the lateral lobes of the cerebellum, like that of one of the hemispheres of the brain, gives rise to paralysis of the opposite side of the body, we should, *a priori,* have expected that hemorrhage of the *left* lobe, for example, of this organ, and of the *right* hemisphere of the brain, occurring together, would have given rise to general paralysis, or of both sides of the body. Such, however, as has been observed by Andral, is not the case; for the paralysis is found to exist on that side of the body only opposite to the hemisphere of the brain which is the seat of the effusion, the other side remaining unaffected by the effusion in the "cerebellum."

The following is the case to which we referred above:

"The most remarkable of these cases occurred in a stout man, a porter, of middle age, who stumbled in the street from the effects of liquor, and struck his head against the pavement. He got up immediately, and walked home. A few days after, he felt himself less capable of making considerable exertion than formerly: he could not, as before the accident, raise a heavy load, or walk steadily under a heavy burden. This state increased, but not to such a degree as to prevent him from continuing his daily occupation for three weeks, at which period he presented himself at the Hôtel Dieu of Lyons. His general health was good; his intellectual faculties were entire; he walked and expressed himself with perfect freedom; the pulse and temperature of the skin were natural. Under these circumstances the receiving physician refused to admit him. Next day he was brought to the hospital in a state of complete coma, with stertorous breathing, and general paralysis, and died during the day. We found that he had gone home, and complained to his wife that he had been refused admission to the hospital because he had no fever; and that she, in order to remove this objection, had administered to him nearly a quart of hot wine, containing a quantity of pepper. The result of the autopsy was interesting: at least six ounces of blood covered the superior half of the surface of the brain, but was separated from it by the arachnoid, between which and the dura mater it was enclosed. The blood was partly fluid and partly coagulated, red or almost black; and a false membrane, of the thickness of card-paper, lined the internal surface of the dura mater in contact with the blood, presenting in several parts of its extent numerous vessels of new formation. The only lesion observed in the brain itself was congestion of its venous system.

"These appearances afford a most satisfactory explanation of the apparent anomaly observed in this interesting case, viz. the absence of paralysis during the existence of an extensive effusion of blood within the cranium, as well as of the cause of the general paralysis and death which supervened. The state of the blood, and the presence of a false membrane, mark the duration of the hemorrhage, and its progressive nature. The slow and gradual effusion of the blood in this case had allowed the circulation of the brain to accommodate itself to the presence of so much additional fluid, thereby obviating the effects of pressure. So long, in fact, as the quantity of the blood circulating in the brain was not increased beyond the limits within which it was compatible with the presence of the blood that was effused, none of the usual effects of compression were observed; but, so soon as the stimulating potion which the unfortunate patient swallowed had, by its effects on the general circulation, and probably also on that of the brain, occasioned an influx of blood towards this organ, compression must then have taken place, as certainly as the general paralysis by which it was almost immediately followed. The slow development of chronic abscess within the substance of the brain, and the gradual accumulation of water in its ventricles, without any accompanying increase of the parietes of the cranium, are lesions perfectly similar in their nature to the former, as regards the law of pressure in the production of paralysis, and which law may be thus generally expressed, and applied to the disturbance of any special function from this cause,—that the disturbance of the functions of an organ from pressure is in the direct ratio of the rapidity with which the compressing cause operates."

Dr. Carswell is of opinion that " the *obstruction* of natural passages by the effused blood is an occurrence which leads to no important modification of function, except in pulmonary hemorrhage." There are cases, however, on record, in which an obstruction of the urethra by a clot of blood has given rise to complete retention of urine; and such cases are the more distressing as the cause is by no means obvious. When ascertained, the obstruction is easily removed by the injection of warm water.

We must content ourselves with one more extract, on hemorrhage in the digestive organs.

" The physical characters of hemorrhage of these organs which require description are few in number, and are referable to the colour, consistence, and quantity of the effused blood. The blood effused into the stomach and intestines is seldom found to present its *natural red colour*, either when thrown out from these organs, or when contained in them after death. It has often acquired a dark purple, and still more frequently a deep brown tint, resembling bistre, or the blackness of soot. The dark brown and sooty discolourations of the blood may always be regarded as the result of the action of an acid chemical agent, formed in the digestive organs, on the effused blood, except in those cases in which they are produced by the introduction of an acid poison. Hence we may conclude, that the diseases called *black vomit* and *melæna* are mere modifications of gastric and intestinal hemorrhage, the black colour being an accidental circumstance of no importance, and derived from the chemical action of the acid product on the blood previous to its evacuation."

The fifth figure of the fourth Plate is a very instructive one, representing the morbid anatomy of the parts which give rise to hemorrhoids. According to our author, there are two forms of this disease,—the one depending on dilatation of the veins of the rectum, and the other on a transformation of the dense cellular tissue of the margin of the anus into erectile tissue.

The plates are, as usual, models of the most finished excellence; and the text is so good, that we could wish to see it published in a separate form, for the benefit of those who may be prevented from purchasing the work as it stands by what Walter Scott calls *impecuniosity.*

